# Annotations

**Published:** 1910-10-29

**Authors:** 


### Annotations.

### Man, Woman, and Athletics.

During the present month a match at golf between a leading amateur of either sex has attracted a good deal of attention, and will no doubt be referred to in future discussions as to the relative physical powers of men and women. Coming, as it does, soon after an estimate by an experienced observer of their relative fitness for the pursuit of medicine, and fairly recent to the expression of much less frank opinions in a House of Commons' debate, it may well be of interest to others than sportsmen pure and simple. The contest was a very close one, and the lady received a substantial start. It may be regarded as proved then that the masculine capacity for this game exceeds the feminine considerably. On the other hand, Miss Leitch displayed great tenacity, and clearly had none the worst of it as regards moral qualifications. Mr. Roughton's opinions, to which we have referred, thus receive (as far as generalising from a single case warrants) at once confirmation and disproof. His tenets were based on the teaching for which Darwin and, in this country, Havelock Ellis are responsible, namely, that in muscular power and high grades of intellectual ability woman is the inferior, and especially also in number and variety of departures from the normal, as exemplified in genius, idiocy, and rare forms of disease. Of this view (hotly as Professor Pearson has contested the latter part of it) there are very strong reasons in proof, although of course points can be made against it. In connection with athletics we do not remember having ever seen mentioned the proficiency of women in distance swimming. In a recent important long-distance event on level terms a woman finished very near the head of a large field of men—a fact for which the thickness of the subcutaneous fascia in females, giving buoyancy and preventing loss of heat, is perhaps responsible. Again, little as one woman may differ from another on the average, there do exist wide individual variations. Plato was not speaking at random when in his "Republic" he advised that the warriors' wives should fight alongside their husbands.

### Train Lavatories.

The disgraceful arrangements still obtaining on many of our railway lines with regard to lavatory accommodation on the long-distance trains have often been the subject of complaint with passengers, but it is high time that some organised protest was made against the reckless manner in which the average railway company flouts all principles of personal hygiene. The London and Brighton line is one of the worst offenders in this respect, and the London and South Western, especially in its boat trains that run from Waterloo to Southampton, is equally lax in looking after the comfort of passengers. No train that runs without a stop for a longer distance than half an hour should be allowed to be unprovided with lavatory accommodation. If no remedy exists at law for the inconvenience and discomfort to which passengers are often put owing to the absence of all provision for water-closets and urinals on the trains themselves, it should not be difficult to secure a redress of existing grievances under the powers conferred on local authorities at the various stations where trains stop. It is disgraceful to allow the present system of apathy and callous disregard of the convenience and health of passengers to continue. We are fully aware of the fact that our third-class railway accommodation on many of the lines is abominable. A series of glycerine-plate cultures made in a carriage of any of the more or less—from a sanitary point of view—badly managed railways would yield most interesting bacteriological crops, and we are by no means sure whether passengers travelling in such carriages would not be able to substantiate a claim for damages in case they develop any specific disease. Nor are we confident that an action could not be supported on the ground of injury to health arising out of the non-provision of lavatory accommodation.

### The Local Government Board Report.

In the first part of the Blue-book issued by the Local Government Board and dealing with the work of that body during 1909 under the Poor Law, Unemployed Workmen, and Old Age Pensions Acts, there are certain items of interest to the medical profession. Thus with respect to outdoor medical relief the total number of orders issued was 115,623, a decrease of forty-four on the figures for 1908. The average number of orders per medical officer was 756, and the average remuneration per order was 3s. 1½d.; if fees for midwifery attendances be included, the average remuneration is raised by 3d. Now Poor Law attendance in rural districts often means a journey of several miles; it also includes the provision of medicines and dressings; and one order may involve (and quite frequently does so) a long series of visits, not uncommonly to cases of infectious disease. It is apparent, then, that a Poor Law medical officership is no sinecure and is very grossly underpaid. There is no doubt that most of such officers do their duty very conscientiously to the best of their abilities, and that many of them make nothing whatever out of it. No other profession but ours would continue to work for the State on such sweated terms; but while the organisation of the profession remains so defective it seems hopeless to expect any improvement. Turning to other sections of the report it is interesting to note that since 1908 the average duration of treatment under *x*-rays for ringworm has fallen from five to three and a half months per case. This reduction is most satisfactory, but it is apparent that even the newest and most approved remedy for this refractory disease takes some time to effect cure. Another table shows the number of dismissals (including resignations to avoid dismissal) of Poor Law officers. We note with satisfaction that among 4,781 medical officers there were but three such cases, as against seven out of 821 masters of workhouses and superintendents and seven out of 1,773 relieving officers.

